# Direct interaction between centralspindlin and PRC1 reinforces mechanical resilience of the central spindle

**DOI:** 10.1038/ncomms8290

**Published:** 2015-06-19

**Authors:** Kian-Yong Lee, Behrooz Esmaeili, Ben Zealley, Masanori Mishima

**Affiliations:** 1 Wellcome Trust/Cancer Research UK Gurdon Institute, University of Cambridge, Cambridge CB2 1QN, UK; 2 Division of Biomedical Cell Biology, Warwick Medical School, University of Warwick, Coventry CV4 7AL, UK

## Abstract

During animal cell division, the central spindle, an anti-parallel microtubule bundle structure formed between segregating chromosomes during anaphase, cooperates with astral microtubules to position the cleavage furrow. Because the central spindle is the only structure linking the two halves of the mitotic spindle, it is under mechanical tension from dynein-generated cortical pulling forces, which determine spindle positioning and drive chromosome segregation through spindle elongation. The central spindle should be flexible enough for efficient chromosome segregation while maintaining its structural integrity for reliable cytokinesis. How the cell balances these potentially conflicting requirements is poorly understood. Here, we demonstrate that the central spindle in *C. elegans* embryos has a resilient mechanism for recovery from perturbations by excess tension derived from cortical pulling forces. This mechanism involves the direct interaction of two different types of conserved microtubule bundlers that are crucial for central spindle formation, PRC1 and centralspindlin.

## Abstract (web summary)

The central spindle is an anti parallel bundle of microtubules that forms between segregating chromosomes and links the two halves of the mitotic spindle. Lee *et al.* reveal that interaction between two microtubule bundling proteins at the central spindle confers robustness to cortical pulling forces.

## Graphical abstract

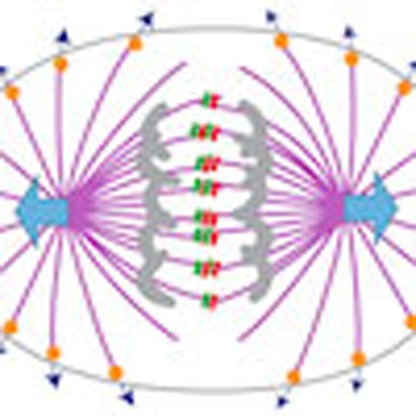

Microtubule bundle structures play crucial roles in animal cell cytokinesis. During anaphase, the central spindle is formed between segregating chromosomes, and, in cooperation with astral microtubules, it determines the position of the cleavage furrow by forming a zone of active Rho GTPase, a master regulator of contractile ring formation^1^. It also serves as a precursor to the midbody, which recruits factors crucial for the final separation of two daughter cells by membrane abscission^2^,^3^. Dynein at the cell cortex generates ‘cortical pulling force', a mechanical force that pulls astral microtubules towards the cell cortex^4^,^5^. As a consequence, the spindle poles are pulled outwards. This force determines spindle positioning and drives chromosome segregation through elongation of the pole-to-pole distance (anaphase B)^4^,^6^,^7^,^8^,^9^,^10^. After anaphase onset, when sister chromatid cohesion has dissolved, the central spindle becomes the only structure that links the two halves of the mitotic spindle. Thus, it is under mechanical tension from the cortical pulling force. How the central spindle maintains its structural integrity under tension while allowing anaphase B pole-to-pole elongation to occur is not well understood.

Molecules important for the formation and function of the central spindle form a protein–protein interaction network that includes two different microtubule-bundling factors, centralspindlin and PRC1 (refs 11, 12). Centralspindlin is a 2:2 heterotetramer of a kinesin-6 subunit MKLP1 (MKLP1/KIF23 in mammals and ZEN-4 in *Caenorhabditis elegans*) and a non-motor subunit CYK4 containing a GTPase-activating protein (GAP) domain for Rho-family GTPases (MgcRacGAP/RACGAP1 in mammals and CYK-4 in *C. elegans*)^13^,^14^. PRC1 is the metazoan orthologue of yeast Ase1 and plant MAP65 (refs 15, 16). Both centralspindlin and PRC1 show microtubule-bundling activities *in vitro* and are required for proper formation of the central spindle *in vivo*. They co-localize to the centre of the central spindle and midbody, where the plus ends of microtubules are bundled in an interdigitating manner^17^. As important hubs of the protein–protein interaction network, both of these proteins recruit various other factors to the centre of the central spindle and midbody. For example, KIF4 kinesin, a suppressor of microtubule polymerization dynamics, is recruited to the central spindle via interaction with PRC1 and limits the length of the anti-parallel overlap^18^,^19^,^20^,^21^. ECT2, a major activator of Rho during cytokinesis, accumulates at the spindle midzone through interaction with CYK4 (refs 22, 23, 24, 25, 26, 27) and forms an equatorial zone of active Rho^28^. Interestingly, a direct interaction between PRC1 and CYK4 has been reported in mammalian cells^29^. However, the role of the interaction between the two different types of microtubule bundling proteins in central spindle formation remains unclear. Here, we study the response of the central spindle to mechanical perturbation and find that the interaction between centralspindlin and PRC1 plays an important role in the mechanical robustness of this microtubule bundle structure that is critical for cytokinesis.

## Results

### Resilience of central spindle under mechanical perturbation

**Figure 1:**
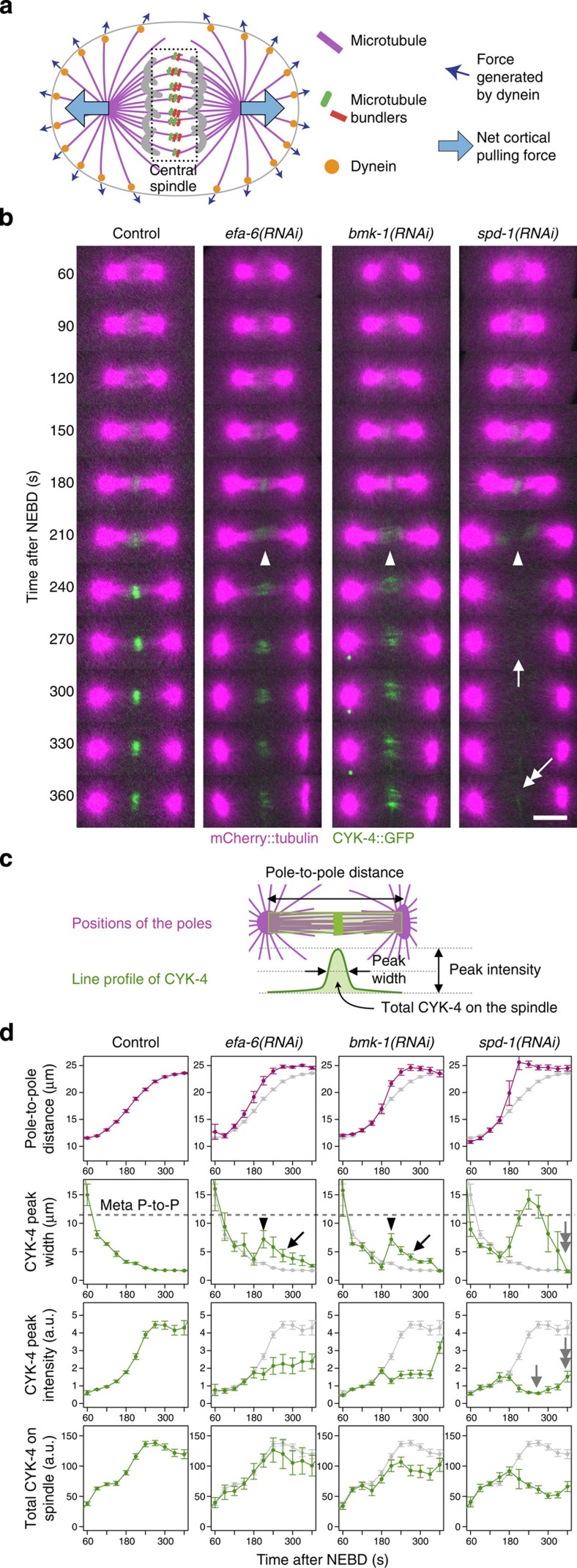
Resilience of central spindle against excess tension. The central spindle in *C. elegans* can recover from near disruption during the first embryonic division. (**a**) Schematic of an animal cell in anaphase. The central spindle is under mechanical tension from cortical pulling forces. (**b**) Spinning disk confocal time-lapse images of embryos expressing mCherry::tubulin (magenta) and a major organizer of the central spindle, CYK-4::GFP (green), after the depletion of EFA-6, BMK-1 and SPD-1 by RNAi. The arrowheads indicate the sudden broadening of CYK-4 midzone accumulation associated with the mid-anaphase acceleration of pole-to-pole elongation caused by the depletion of these molecules. The central spindle was not completely broken in *efa-6(RNAi)* (enhanced outward pulling force) or in *bmk-1(RNAi)* (less drag against the pulling force) embryos, and CYK-4 accumulation was later restored. This recovery did not occur in embryos depleted of SPD-1/PRC1, another major central spindle organizer. The midzone accumulation of CYK-4 was largely lost (arrow), although weak accumulation via the furrow-dependent pathway^35^ was observed later (double-headed arrow). Scale bar, 10 μm. (**c**, **d**) Quantitative analysis of morphological changes in the mitotic spindle. The positions of the spindle poles were determined as the peak mCherry::tubulin signals, and the distance between them (pole-to-pole distance) is plotted in magenta. The intensity of CYK-4::GFP in individual embryos was standardized using a uniform cytoplasmic signal during the pre-mitotic stage. Line profiles of 4.4 μm in width between the two poles were measured for each time point, and the width at half-maximum (CYK-4 peak width), height (CYK-4 peak intensity) and area (total CYK-4 on spindle) of the peak were measured and are plotted in green. The dotted lines on the graphs of CYK-4 peak width indicate the pole-to-pole distance during metaphase. In the *efa-6(RNAi)*, *bmk-1(RNAi)* and *spd-1(RNAi)* panels, the values for the control are also plotted in grey. Recovery after near disruption in *efa-6(RNAi)* and *bmk-1(RNAi)* embryos was detected as gradual tightening of the CYK-4 peak width (arrows) after sudden widening (arrowheads). The grey arrows indicate the loss of CYK-4 accumulation in *spd-1(RNAi)* embryos and the central spindle-independent late recruitment. The mean and standard error were determined from 33, 7, 6 and 6 embryos for the control, *efa-6(RNAi)*, *bmk-1(RNAi)* and *spd-1(RNAi)*, respectively.

To examine the response of the central spindle to increased pulling forces (Fig. 1a), we generated transgenic *C. elegans* strains expressing mCherry-tagged tubulin and green fluorescent protein (GFP)-tagged CYK-4. During the first mitotic division of control embryos, the bipolar spindle formed promptly following nuclear envelope breakdown (NEBD, time=0). NEBD was monitored by the loss of exclusion of the tubulin signal from pronuclei (Fig. 1b and Supplementary Fig. 1 for grayscale images covering the whole embryos). Approximately 120–150 s following NEBD, the distance between the two spindle poles (Fig. 1c) started to increase, indicating the onset of anaphase, as previously reported^30^ (Fig. 1d). At the same time, CYK-4 began to accumulate at the spindle midzone (Fig. 1b, control), where the plus ends of the two sets of interpolar microtubules from the two spindle poles form an interdigitating overlap, as confirmed by an accelerated increase in the peak intensity (Fig. 1d, control and Supplementary Fig. 2a for examples of the line profiling of CYK-4::GFP intensity). Within the next 30–60 s, the central spindle was established, with CYK-4 enriching rapidly in the central overlap zone and remaining there for ∼300 s until the cleavage furrow fully ingressed and the central spindle was compacted to form the midbody.

EFA-6 is a negative regulator of the dynein-based cortical pulling force^31^. In embryos depleted of EFA-6, pole-to-pole elongation was accelerated (from 80 nm s^−1^ in control embryos to 120 nm s^−1^; Fig. 1d, *efa-6(RNAi)*), as expected. In these embryos, CYK-4 accumulated normally until 180 s after NEBD (Fig. 1b,d, *efa-6(RNAi)*), when the peak corresponding to its accumulation suddenly broadened (Fig. 1b,d, arrowheads). Thereafter, the total amount of CYK-4 detected between the two poles continued to increase, and the tight localization of CYK-4 fluorescence at the midzone was gradually restored (Fig. 1d, black arrows in the ‘CYK-4 peak width' panels). A similar pattern of CYK-4 accumulation was observed in the embryos depleted of the kinesin-5 BMK-1, which has been reported to function as a brake during spindle elongation in *C. elegans* embryos^32^ and other cell types^33^,^34^ (Fig. 1b,d, *bmk-1(RNAi)*). These data indicate that the central spindle has a resilient recovery mechanism that acts against perturbations that cause excessive pole-to-pole separation.

In contrast, in embryos depleted of SPD-1, the *C. elegans* orthologue of PRC1, the spindle was broken into two halves, and the accumulation of CYK-4 at the midzone dropped drastically (Fig. 1b,d, arrow in *spd-1(RNAi)* and Supplementary Fig. 2b and c)^35^. Once it was lost from the spindle midzone, CYK-4 could not re-accumulate there, although much later, it localized to the midbody via the cortical/astral route independent of the central spindle (Fig. 1b,d, double-headed arrows in *spd-1(RNAi)* panels)^35^,^36^. Reciprocally, the dependency of SPD-1 midzone localization on centralspindlin has also been reported^35^, implying that the cooperative interaction between the two different types of microtubule bundling proteins might be important for the mechanical resilience of the central spindle.

### Direct interaction between PRC1 and centralspindlin

**Figure 2:**
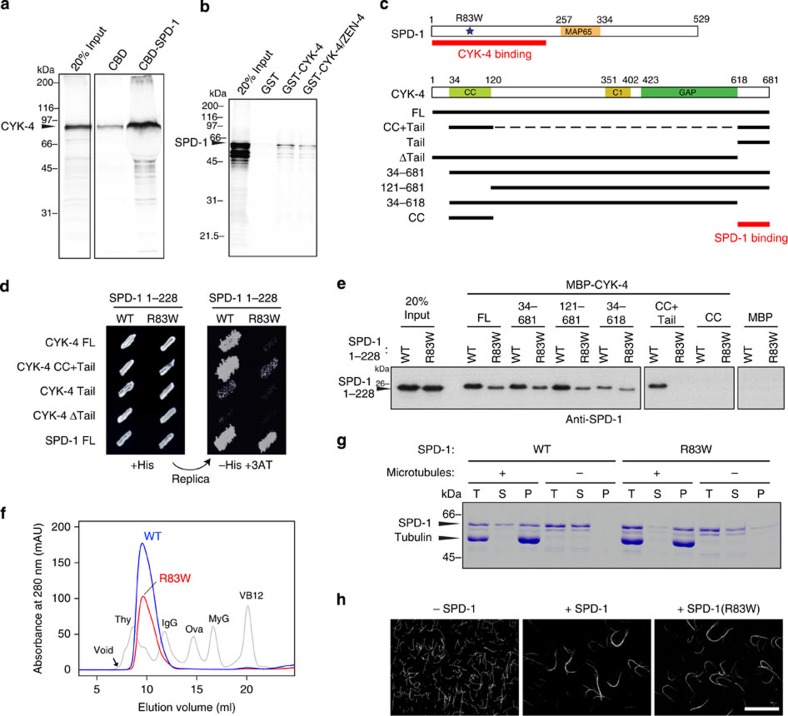
Physical interaction between SPD-1 and CYK-4 sensitive to SPD-1 R83W mutation. (**a**) *In vitro* translated full-length CYK-4 was pulled down by full-length SPD-1 immobilized on chitin beads via a chitin-binding domain (CBD) tag. (**b**) *In vitro* translated full-length SPD-1 was pulled down by full-length CYK-4 or the centralspindlin holocomplex (CYK-4/ZEN-4) immobilized on glutathione-Sepharose beads via a glutathione-*S*-transferase (GST) tag. (**c**) Schematic drawings of SPD-1 and CYK-4. R83W indicates the mutation found in the *spd-1(oj5)* mutant exhibiting central spindle defects. (**d**) Yeast 2-hybrid assay of the indicated combinations of bait and prey. Growth on histidine-deficient medium containing 3-amino-1,2,4-triazole (–His+3AT) indicates a positive interaction between the bait and prey. (**e**) SPD-1 1-228 fragment with or without the R83W mutation (WT: wild type) was pulled down by CYK-4 constructs expressed as fusion proteins with maltose-binding protein (MBP) and detected with an anti-SPD-1 antibody. The CYK-4 tail region is necessary and, if dimerized, sufficient for efficient binding. (**f**) The R83W mutation does not affect the mobility of the SPD-1 full-length protein in Superdex 200 size-exclusion chromatography (blue: wild type; red: R83W). The elution profile of a mixture of standard proteins (Thy, thyroglobulin; IgG, gamma globulin; Ova, ovalbumin; MyG, myoglobin; VB12, vitamin B12) is shown in grey. (**g**, **h**) The R83W mutation does not affect the interaction of SPD-1 with microtubules. (**g**) Wild-type and R83W SPD-1 were incubated with microtubules or control buffer and sedimented by ultracentrifugation. P, pellet; S, supernatant; T, total. Increased recovery in the pellet in the presence of microtubules indicates the co-precipitation of SPD-1 with the microtubules. (**h**) Microtubules were incubated with SPD-1 with or without the R83W mutation and visualized by immunofluorescence following fixation. Scale bar, 20 μm.

A direct interaction between human orthologues of SPD-1 and CYK-4 has been reported^29^, and a physical interaction between Ase1, a yeast PRC1 homologue, and Klp9, a kinesin that is distantly related to ZEN-4/MKLP1 (although more closely to MKLP2), has also been shown^37^. We therefore examined whether SPD-1 and CYK-4 physically interact by *in vitro* pull-down and yeast two-hybrid assays. *In vitro* translated full-length CYK-4 was pulled down by beads coated with bacterially expressed full-length SPD-1 (Fig. 2a and Supplementary Fig. 3a). Inversely, SPD-1 was bound to beads coated with CYK-4 as well as the CYK-4/ZEN-4 centralspindlin holocomplex (Fig. 2b and Supplementary Fig. 3b). This interaction was also observed in yeast two-hybrid assays between CYK-4 and the N-terminal fragment of SPD-1 and was drastically weakened by deletion of the C-terminal tail of CYK-4 (Fig. 2c,d CYK-4 ΔTail), which is dispensable for both centralspindlin complex assembly and microtubule bundling^13^,^38^. In contrast, the CYK-4 C-terminal tail fragment alone showed an interaction with SPD-1 that was enhanced by dimerization by adding back the coiled coil (Fig. 2c and d, CYK-4 Tail and CC+Tail). A similar requirement of the C-terminal tail of CYK-4 was also observed in pull-down assays (Fig. 2e and Supplementary Fig. 4). SPD-1 1–228 was robustly pulled down by CYK-4 fragments containing the C-terminal tail region (FL, 34–681, 121–681 and CC+Tail) but much more weakly by fragments lacking the C-terminal tail (34-618 and CC). These data indicate that the SPD-1–CYK-4 interaction is mediated mainly by the N-terminal region of SPD-1 and the C-terminal tail of CYK-4.

The *spd-1* gene was originally isolated in a forward genetic screen for temperature-sensitive cell division defects^39^. Interestingly, the *oj5* allele isolated in this screen as the cause of central spindle defects has been shown to be an R83W mutation^35^, which lies within the CYK-4-binding region of SPD-1 defined above. We tested whether this mutation affected the SPD-1–CYK-4 interaction and found that it was drastically weakened in both yeast two-hybrid (Fig. 2d) and *in vitro* pull-down assays (Fig. 2e and Supplementary Fig. 4). Importantly, however, this mutation did not affect other known properties of PRC1, such as dimerization/oligomerization^40^,^41^ and microtubule bundling. In yeast two-hybrid assays, the interaction between SPD-1 expressed as bait and SPD-1 as prey was not affected by the R83W mutation (Fig. 2d, bottom row). Consistently, this mutation did not affect the mobility of full-length recombinant SPD-1 in size exclusion chromatography (Fig. 2f). In addition, SPD-1 with the R83W mutation could bind microtubules (Fig. 2g) and bundle microtubules (Fig. 2h) in solution as efficiently as its wild-type counterpart. These data indicate that the R83W mutation specifically affects the interaction between SPD-1 and CYK-4, although we cannot formally exclude possible influences on as-yet-unknown functions of SPD-1/PRC1. These findings strongly suggest that the central spindle defects observed in *spd-1(oj5)* mutants are caused by the defective binding between PRC1 and centralspindlin, the two major microtubule crosslinking/bundling proteins of the central spindle.

### Rupture of central spindle caused by CYK-4 tail mutations

**Figure 3:**
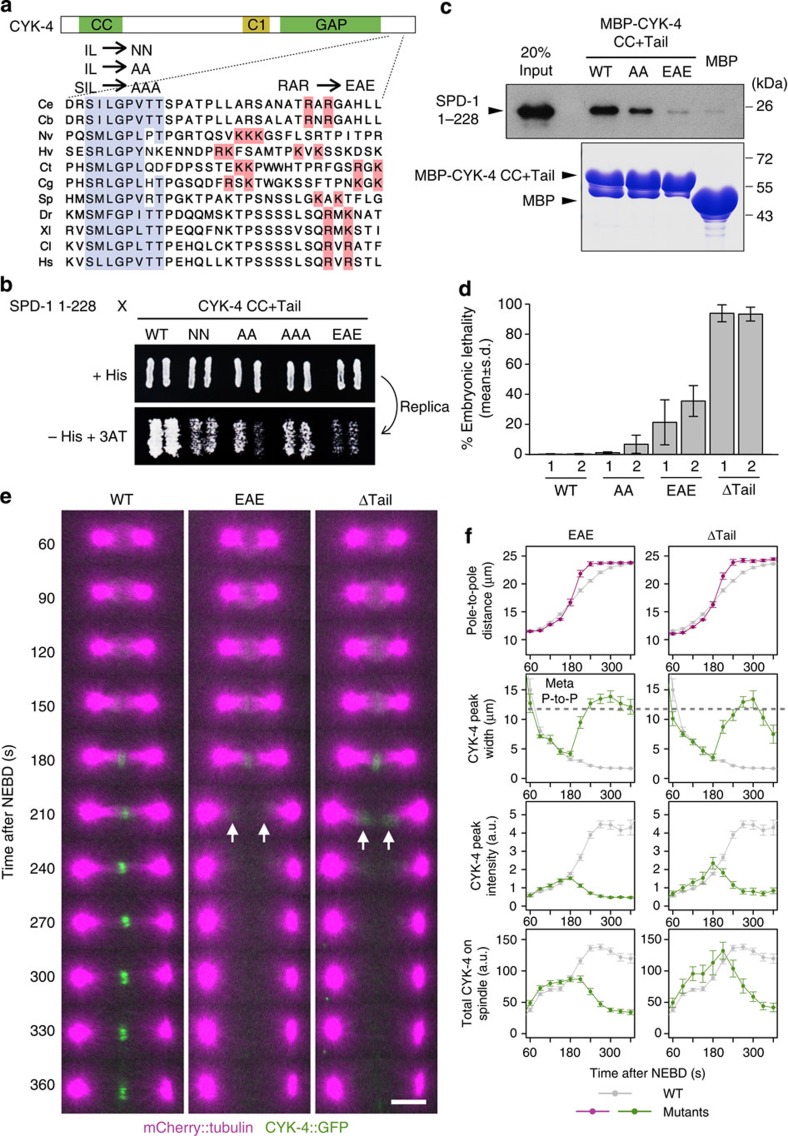
Mutations in the C-terminal tail of CYK-4 affect the SPD-1–CYK-4 interaction and cause mid-anaphase spindle rupture. (**a**) An alignment of the CYK-4 C-terminal tail region, which corresponds to amino acids 644–675 of the *C. elegans* protein. The conserved motifs with the mutations assessed are highlighted. Ce, *C. elegans*; Cb, *C. briggsae* (nematode); Nv, *N. vectensis* (sea anemone); Hv, *H. vulgaris* (hydra); Ct, *C. teleta* (annelid); Cg, *C. gigas* (oyster); Sp, *S. purpuratus* (sea urchin); Dr, *D. rerio* (zebrafish); Xl, *X. laevis* (frog); Cl, *C. livia* (bird) and Hs, *H. sapiens.* (**b**) Yeast two-hybrid assay assessing the effects of mutations within the above conserved motifs. (**c**) An SPD-1 1-228 fragment was evaluated for *in vitro* binding with wild-type or mutant (AA and EAE) CYK-4 constructs immobilized on beads via a maltose-binding protein (MBP) tag (bottom panel, Coomassie staining) and detected with an anti-SPD-1 antibody (top panel). (**d**) Rescue of the embryonic lethality of *cyk-4* null embryos by the indicated *cyk-4::gfp* transgenes. For each strain (two independent strains each for wild-type, AA, EAE and ΔTail), at least 690 embryos produced by seven hermaphrodites homozygous for the *cyk-4* null allele and the *cyk-4::gfp* transgene were scored. (**e**,**f**) Spinning disk confocal time-lapse imaging of embryos expressing mCherry::tubulin and the indicated *cyk-4::gfp* transgene in the null background for the *cyk-4* endogenous locus (*n*=25 and 23 for EAE and ΔTail, respectively). Mid-anaphase spindle rupture was observed in the mutants defective for the SPD-1–CYK-4 interaction (*t*=210 s). No recovery of the midzone accumulation of CYK-4 mutants was observed, although in some embryos, the transient association of mutant CYK-4 near the microtubule plus ends was detected (arrows). Scale bar, 10 μm. In **f**, the data for the mutants are displayed in colour, whereas those for the wild-type control (same as in Fig. 1; *n*=33) are plotted in grey.

To further assess the physiological importance of the interaction between SPD-1 and CYK-4, we introduced point mutations in CYK-4 that affected its interaction with SPD-1. Sequence comparison of the C-terminal tails of CYK-4 orthologues revealed the presence of two motifs, SILGPVTT and K/R-X-K/R, which have been widely conserved throughout metazoan evolution (Fig. 3a and Supplementary Fig. 5). Mutations in these motifs weakened the SPD-1–CYK-4 interaction, either moderately (IL to NN, IL to AA and SIL to AAA) or more severely (RAR to EAE; Fig. 3b). Similar effects were also observed in an *in vitro* pull-down assay (Fig. 3c). We generated transgenic animals carrying these mutations (AA and EAE) as well as a deletion of the tail domain (ΔTail) in a *cyk-4::gfp* transgene and expressed the transgenes in a strain in which endogenous *cyk-4* had been deleted. Although the wild-type transgene efficiently suppressed the embryonic lethality caused by the deletion of endogenous *cyk-4*, embryos expressing the mutant transgenes showed different levels of lethality, according to the severity of the SPD-1-binding defect (Fig. 3d). Although the mild AA mutation caused mild lethality, the more severe EAE mutation caused much higher lethality. The ΔTail mutation resulted in the most severe phenotype (>90% lethality). This close correlation between the *in vitro* and *in vivo* phenotypes clearly shows that the SPD-1–CYK-4 interaction plays a crucial role in early *C. elegans* development.

Next, we examined the influences of the defective SPD-1–CYK-4 interaction on cell division, especially on the robustness of the central spindle. The fluorescence intensities of uniform cytoplasmic CYK-4::GFP during the pre-mitotic stage confirmed that the protein expression level was not affected by the EAE and ΔTail mutations (Supplementary Fig. 6). Consistent with the normal behaviour of CYK-4 in *spd-1(RNAi)* embryos up to mid-anaphase (NEBD ∼180 s), in the EAE and ΔTail mutant embryos, the bipolar spindle formed normally, and mutant CYK-4 began to accumulate at the spindle midzone at the same time as in wild-type embryos (Fig. 3e,f). However, once it formed, the midzone microtubule bundle was disrupted within 30–60 s. As a consequence, the anaphase spindle was split into two half-spindles, and the poles moved rapidly apart. At the same time, accumulated mutant CYK-4 was lost, detected as a rapid broadening of the peak width and a gradual decrease in the peak intensity (Fig. 3f) of CYK-4::GFP fluorescence, indicating that in some embryos, mutant CYK-4 was retained near the tips of the broken half-spindles for a while (Fig. 3e, arrows). These observations clearly indicate that the SPD-1–CYK-4 interaction is necessary for the robust maintenance of the central spindle after its initial formation.

### Suppression of spindle rupture by reduction of tension

**Figure 4:**
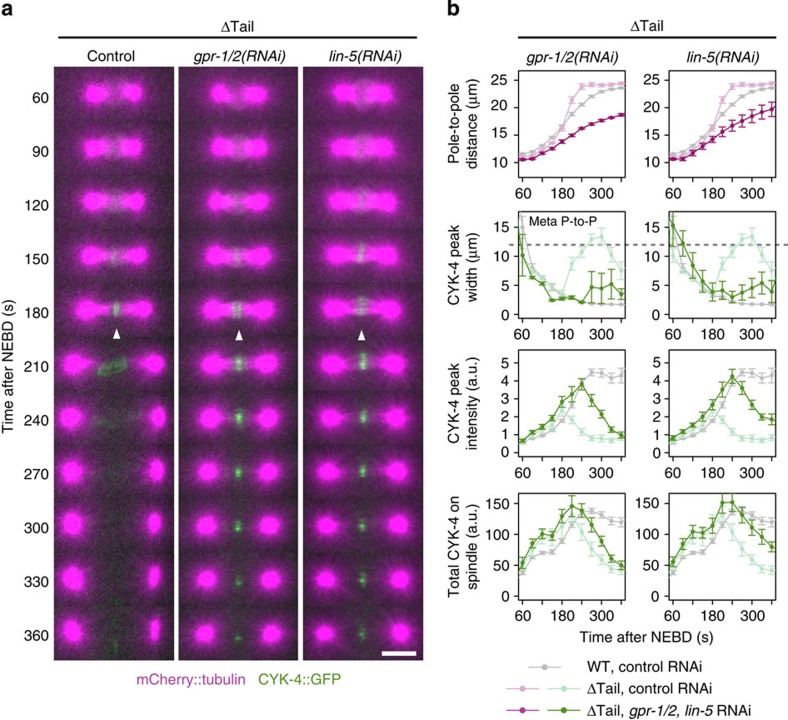
Mid-anaphase spindle rupture in the *cyk-4* mutants defective for the SPD-1–CYK-4 interaction is suppressed by reduction of the cortical pulling force. (**a**,**b**) Time-lapse imaging of the *cyk-4* ΔTail mutant embryos depleted of an activator of the cortical pulling force, GPR-1/2 or LIN-5 (*n*=6 and 11, respectively). Although the central spindle was broken in control embryos (same as in Fig. 3, *n*=23), in *gpr-1/2(RNAi)* or *lin-5(RNAi)* embryos, the central spindle and the midzone accumulation of the CYK-4 mutant (arrowheads) were stably maintained. Scale bar, 10 μm. In **b**, the data for the reduced pulling force (*gpr-1/2(RNAi)* or *lin-5(RNAi)*) are plotted in bold colour, whereas those for the ΔTail mutant and wild-type embryos with unperturbed cortical pulling forces (control) are depicted in pale colour and grey, respectively. Similar effects of the reduction of the cortical pulling force were observed in EAE mutant embryos (Supplementary Fig. 7).

Because enhancement of the cortical pulling force by *efa-6(RNAi)* caused pole separation to accelerate (Fig. 1), we hypothesized that spindle breakage in the absence of the SPD-1–CYK-4 interaction might be suppressed if cortical pulling forces were reduced by the depletion of cortical activators of dynein^5^,^42^ (Fig. 4). As previously reported, depletion of the GoLoco domain protein GPR-1/2 or NuMA-related LIN-5 slowed pole-to-pole separation to ∼40–50 nm s^−1^ (Fig. 4a,b), indicating a reduction in the cortical pulling force^43^,^44^,^45^,^46^. Under these conditions, an apparently normal central spindle was formed in *cyk-4* ΔTail mutant embryos and was maintained thereafter much more stably than in control embryos (Fig. 4a). The peak intensity of CYK-4 ΔTail at late anaphase (240 s after NEBD) was restored to almost 100% of the level of wild-type embryos without RNA interference (RNAi; Fig. 4b). The width of midzone accumulation also became comparable to that of the control, although later, it was slightly broadened (Fig. 4a,b). However, consistent with the role of the G-protein pathway in the regulation of the cortical pulling force in the aster-dependent pathway of cleavage furrow ingression^47^, cytokinesis failure in *cyk-4* ΔTail mutant embryos (13/23) was not suppressed but was rather enhanced by the depletion of GPR-1/2 or LIN-5 (6/6 and 11/11, respectively). Similar restoration of the stability of the central spindle following a reduction in cortical pulling forces was also observed in CYK-4 EAE mutant embryos (Supplementary Fig. 7).

**Figure 5:**
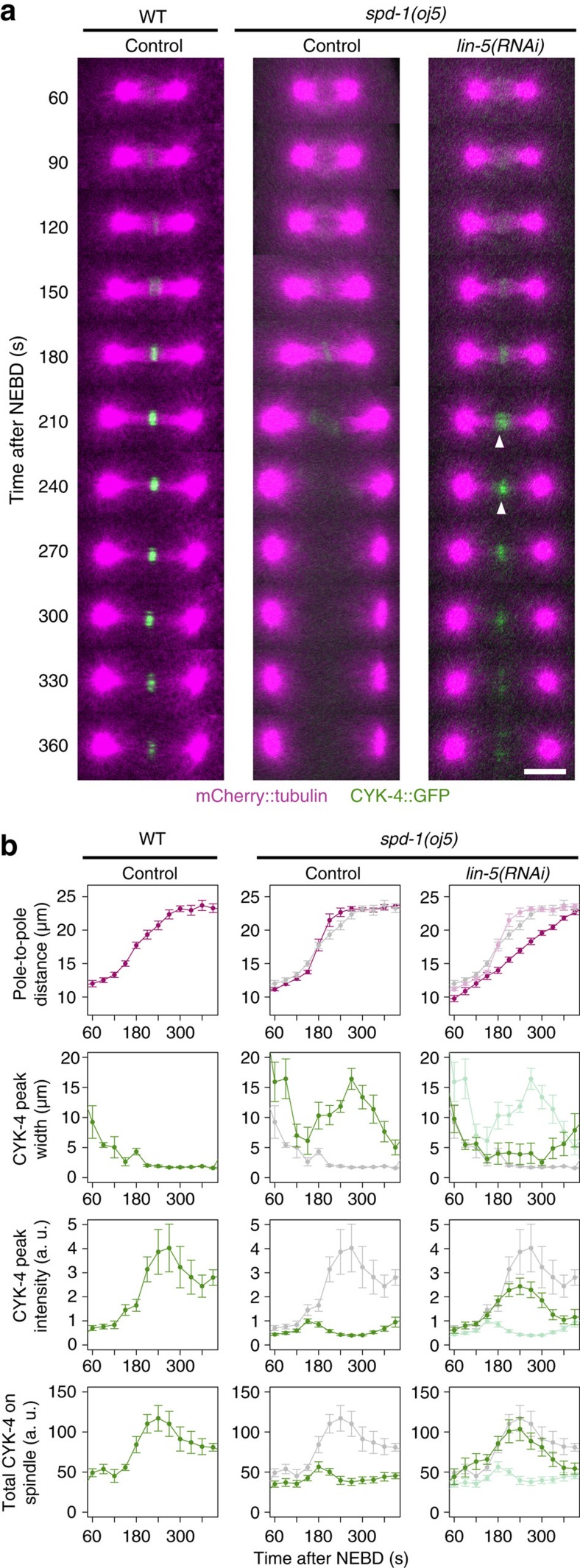
Reduction of the cortical pulling force also suppresses central spindle disruption in *spd-1(oj5)* mutant embryos. (**a**,**b**) Time-lapse imaging of *spd-1(oj5)* embryos expressing mCherry::tubulin and wild-type CYK-4::GFP and depleted of LIN-5 by RNAi (*n*=10). In contrast with the perturbed central spindle in control *spd-1(oj5)* embryos, the central spindle and the midzone accumulation of CYK-4::GFP (arrowheads) were restored when LIN-5 was depleted. Scale bar, 10 μm. In **b**, the data for the reduced pulling force (*lin-5(RNAi)*) are plotted in bold colour, whereas those for the *spd-1(oj5)* mutant (*n*=17) and wild-type (*n*=6) embryos with unperturbed cortical pulling forces (control) are depicted in pale colour and grey, respectively.

To further examine whether the mechanical fragility of the central spindle in the *cyk-4* tail mutants was caused by the lack of SPD-1–CYK-4 interaction or by possible defects in an as-yet-unknown function of the CYK-4 tail, we tested the effect of the reduction of the cortical pulling force in *spd-1(oj5)* embryos (Fig. 5a,b). As previously reported^35^, the central spindle was disrupted in *spd-1(oj5)* embryos, accompanied by the dispersion of transiently accumulated wild-type CYK-4::GFP. In contrast, LIN-5 depletion suppressed the sudden rupture of the central spindle and prolonged the accumulation of CYK-4. These data further indicate that the SPD-1–CYK-4 interaction through the CYK-4 C-terminal tail plays an important role in maintaining the mechanical integrity of the central spindle under tension. This interaction is dispensable for lateral microtubule bundling *per se* and for the tight localization of CYK-4 to the spindle midzone but becomes crucial when the whole spindle is under mechanical tension equivalent to that experienced during the normal asymmetric first cell division.

## Discussion

Here, we have revealed a molecular mechanism for the mechanical resilience of the central spindle, which is key to reconciling the conflicting requirements for efficient cytokinesis versus spindle positioning and chromosome segregation. Interestingly, this mechanism has been implemented into the core machinery of central spindle formation via the direct interaction of two conserved major microtubule bundlers, PRC1 and centralspindlin. The motifs critical for SPD-1 binding in the C-terminal tail of CYK-4 seem to have been widely conserved throughout metazoan evolution^48^, with the exception of arthropods and platyhelminthes (Supplementary Fig. 5). Interestingly, arthropods carry out embryonic cleavage using a unique strategy, and the contribution of the cortical pulling force on anaphase B is limited in *Drosophila* embryos^49^. Because a direct interaction between centralspindlin and PRC1 has been demonstrated in humans and *C. elegans*, a pair of distantly related species, it is reasonable to speculate that the binding between them might also be widely conserved. The interaction between centralspindlin and PRC1 in metazoans might be an evolutionary adaptation to the coupled sliding and polymerization mechanism^50^,^51^, which originally occurred to drive spindle elongation in eukaryotic ancestors, functioning as an ‘engine brake' to counteract excess external forces.

**Figure 6:**
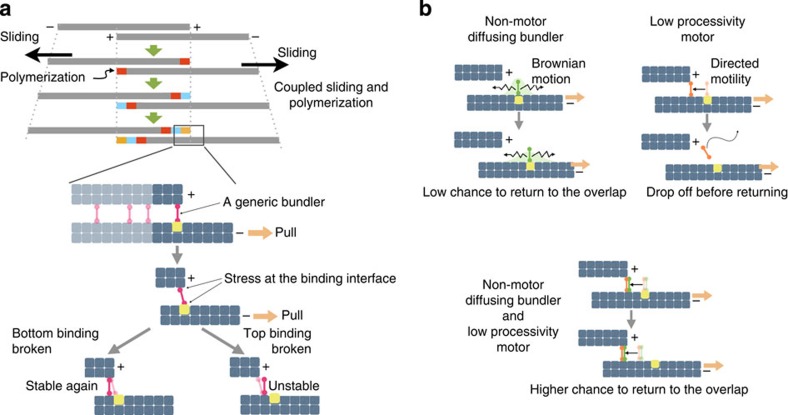
A model for the mechanical robustness of the central spindle, as achieved by interaction between two different types of microtubule-bundling proteins. (**a**) A coupled sliding and polymerization model and the fate of a microtubule bundler at the edge of the sliding overlap zone. Relative sliding between an anti-parallel pair of microtubules by external pulling forces causes stress on a bundler and destabilizes its interaction with microtubules. This stress can be released by the detachment of either one of the microtubule-binding sites from the bundler. When the detachment occurs on the microtubule-binding site that is at the plus end of the microtubule, the bundler cannot return to the overlap zone easily because the site on which it was originally positioned (yellow) moves away due to sliding. (**b**) A non-motor diffusing bundler can remain on a non-bundled microtubule without detachment. However, because the distance that it can move by diffusion is limited, the chance that this molecule can return to the overlap zone is low when rapid sliding is occurring. Even a motor that can move along a microtubule faster than the rate of sliding might fail to return to the overlap zone by dropping off the microtubule. If the two types of microtubule-bundling proteins are associated, they can compensate for each other's limitations and have a greater chance of returning to the overlap zone without detachment and re-establishing crosslinkage.

External pulling force applied to the central spindle accelerates the anti-parallel sliding of microtubule bundles, which would cause constant ‘leakage' of microtubule-bundling proteins from the zone of anti-parallel microtubule overlap into the non-bundled part of the microtubules, where binding of the bundling proteins to them would be less stable (Fig. 6a). We speculate that the interaction between different types of microtubule bundlers might compensate for their limitations, such as the inability of PRC1 to carry out active unidirectional transport^52^,^53^,^54^,^55^ and the low processivity of centralspindlin as a motor, especially when it is not clustered^56^, potentially helping them return to the anti-parallel microtubule overlap (Fig. 6b). In the absence of the PRC1-centralspindlin interaction, slowing anti-parallel sliding would reduce the leakage of bundling proteins from the overlap zone but not completely prevent it. This notion is consistent with the gradual loss of CYK-4 signals observed at the later time points (Figs 4 and 5, ∼300 s after NEBD). This model also predicts that even when the interaction between PRC1 and centralspindlin is intact, if the cortical pulling force is too strong, the central spindle will still be broken. This situation might have occurred in the case of the midzone rupture reported following KLP-7/CeMCAK depletion^4^,^46^. Direct testing of this model would require precise measurement of the distribution of microtubule plus ends and the lengths of anti-parallel overlap as well as the localizations of centralspindlin and PRC1 at higher temporal resolution. In future studies, it will be important to examine how other known protein–protein interactions at the central spindle, such as that between KIF4 chromokinesin and PRC1, which is promoted by Aurora B phosphorylation and suppresses the plus-end dynamics of microtubules^18^,^41^,^53^,^57^,^58^, contribute to the mechanical resilience of the central spindle in cooperation with the PRC1–centralspindlin interaction investigated here.

## Methods

### *C. elegans* strains and culture

The *C. elegans* strains used in this study are listed in Supplementary Table 1 and were maintained at 20 °C, except for strains containing temperature-sensitive *spd-1(oj5)* allele, which were maintained at 15 °C. To generate the *cyk-4::gfp* strains by Mos1-mediated single-copy insertion (MosSCI)^59^, a 4.5-kb *cyk-4* genomic fragment was amplified with a primer pair, 5′-AATCAGGGCCCTCACAAACACAGCACTCGGTC-3′ and 5′-ACTATCCCGGGTACTCACCGGAAACGGAGTC-3′ and cloned into the PspOMI/XmaI site of the pBluescript vector. Following insertion of the *gfp* sequence between the coding region and the stop codon, the BsiWI/XmaI fragment (from 990 bp upstream of the start codon to 1,072 bp downstream of the stop codon) was subcloned into a MosSCI targeting vector, pCFJ178. The point mutations and deletion were introduced with primer pairs, 5′-GCTCTCTGTGATCGTAGCGCTGCTGGACCAGTTACAACATCACC-3′ and 5′-GGTGATGTTGTAACTGGTCCAGCAGCGCTACGATCACAGAGAGC-3′ (‘AA'), 5′-GTCGGCCAACGCGACTGAAGCAGAAGGTGCTCATCTGC-3′ and 5′-GCAGATGAGCACCTTCTGCTTCAGTCGCGTTGGCCGAC-3′ (‘EAE'), and 5′-TGCAGATGTCCCTAGGAATCGTTGCCAATATAC-3′ and 5′-CATCTGCTGGGGTCGATGTTCCACGAT-3′ (‘ΔTail'). The engineered transgenes were confirmed by sequencing and injected into the EG5003 strain for insertion into chromosome IV. Transgene integration was verified by sequencing. mCherry::tubulin strain (JA1559) was a gift from J. Ahringer (Gurdon Institute, UK).

### Embryonic lethality assay

To score the rescue efficiency of *cyk-4* null embryonic lethality by the *cyk-4::gfp* transgenes, the *cyk-4::gfp* MosSCI strains were crossed into the *cyk-4(ok1034)* deletion strain (VC859) to obtain worms homozygous for both *cyk-4(ok1034)* and *cyk-4::gfp,* which were verified by PCR using a pair of primers, 5′-TTATGGACGGTTGTGT-3′ and 5′-GAGACTGTCACCAGGTTG-3′, and a combination of three primers, 5′-GCCGCACACCTTCTCTTTTGATG-3′, 5′-GCAGTACAGAATAATAAAGTGTG-3′ and 5′-GCACGATTTTTGCCATACTACTG-3′, respectively. In the absence of a *cyk-4* transgene, homozygous *cyk-4* deletion (*ok1034*) causes 100% embryonic lethality. The hatching rates of the embryos laid during the first 36 h from late L4 stage by the *cyk-4(ok1034); cyk-4::gfp* homozygous worms were scored 24 h later.

### RNAi

RNAi depletion was performed by feeding L4 hermaphrodites with HT115(DE3) *Escherichia coli* clones expressing double-stranded RNA from L4440 plasmid containing a DNA fragment specific for the target gene^60^. All RNAi clones used in this study (except for that for *spd-1,* which was made by a primer pair, 5′-GCGGATCCATGGCCCGAAGGCACAG-3′ and 5′-CCCAAGCTTTCACAAAAACTGATTTCGTCTCG-3′) were kindly given by J. Ahringer (Gurdon Institute, UK). Control RNAi was conducted using the HT115(DE3) transformed with the empty L4440 vector. Worms were fed on the RNAi plates for 36 h at 20 °C before imaging. For RNAi in temperature-sensitive strains, L4 hermaphrodites were fed for 72 h at 16 °C before imaging.

### Live imaging and quantitative analysis

For live imaging, two or three gravid hermaphrodites were dissected in 2 μl egg salt buffer on a 18 × 18 mm^2^ coverslip to release embryos, inverted onto 2% (w/v )agarose (in 0.5 × egg salt buffer) pad on a slide glass and sealed with petroleum jelly. The slide was taped onto a modified Linkam PE100-ZAL cooling stage connected to a Linkam sPE94 temperature controller set at 22 °C. Newly fertilized embryos were filmed using an Olympus IX81 inverted microscope, with an UPlanSApo 60 × /1.35 objective and a Yokogawa CSU22 spinning disk scanner unit. Every 30 s, a z-series of 21 planes at 1-μm intervals excited at 491 nm (for CYK-4::GFP), followed by another z-series of 21 planes at 1-μm intervals excited at 561 nm (for mCherry::tubulin), were captured with an iXon EMCCD DV885KCS-VP camera (Andor) at 100 ms exposure with no binning, controlled using Metamorph software (Molecular Devices). For the experiments shown in Fig. 5, the embryos were mounted on an agarose pad on the FCS2 cooling device (Bioptechs) connected to a Nano-Therm System (Bioptechs) set at 18 °C and filmed with a Revolution XD system (Andor) equipped with an iXon Ultra 897 EMCCD camera (Andor).

Time-lapse images were analysed using ImageJ (Rasband, W.S., ImageJ, US National Institutes of Health, Bethesda, MD, USA, http://imagej.nih.gov/ij/, 1997–2011) and R (http://www.r-project.org) software. Equal expression of the wild-type and mutant *cyk-4::gfp* transgenes was confirmed by measuring the intensities of the GFP signals (Supplementary Fig. 6). For the mCherry::tubulin channel, the z-slice with the best focus of the two spindle poles was identified for each time frame, and using this slice and the three slices above and below it, an average z-projection was produced. For the CYK-4::GFP channel, the z-slice with a peak signal within a minimum oval defined by the two spindle poles was identified, and a z-projection was produced as described above. To remove the variability between embryos, the fluorescence intensities were standardized according to the total signal levels for each embryo during the pre-mitotic stage. Images for presentation were produced by applying the same colour look-up tables to the standardized images. To quantify the accumulation of CYK-4 at the spindle midzone, a line profile along a spline curve that passed these three points (4.4-μm width, 6-μm z-depth) was obtained and the peak height, the peak width at half maximum and the peak area of the line profile were measured.

### Protein–protein interaction and other biochemical assays

SPD-1 N-terminally tagged with a chitin-binding domain (CBD) was expressed from pCBD-TEV vector in bacteria and purified with chitin beads (New England Biolabs). CYK-4 N-terminally tagged with glutathione *S*-transferase was expressed from pGEX-TEV vector in bacteria and purified with glutathione Sepharose 4B (GE Healthcare). The complex of glutathione *S*-transferase-CYK-4 and CBD-ZEN-4 was expressed from a bicistronic pGEX-6rbs vector in bacteria and purified by sequential binding to chitin beads, elution by TEV protease cleavage of the CBD tag, and binding to glutathione Sepharose 4B. For experiments with radioactive probes, ^35^S-labelled proteins were produced by TNT T7 Coupled Reticulocyte Lysate System (Promega). A volume of 10 μl of the lysates were mixed with 5 μl of beads with immobilized bacterially expressed proteins in 170 μl cold TGMN butter (20 mM Tris-HCl (pH 8.0), 20% (v/v) glycerol, 5 mM MgCl_2_, 150 mM NaCl, 0.1% (v/v) NP-40, 1 mM dithiothreitol, 10 μg ml^−1^ leupeptin, 10 μg ml^−1^ pepstatin and 0.2 mM phenylmethanesulfonylfluoride). For experiments without radioactive probe, purified SPD-1 1–228 fragments were incubated with amylose beads immobilized with fusion proteins of maltose-binding protein and CYK-4 fragments, and detected by western blotting with an anti-SPD-1 antibody (0.1 μg ml^−1^), which was produced in rabbit with bacterially expressed full-length recombinant SPD-1 as an immunogen (Eurogentec) and affinity-purified against SPD-1 191-529.

Yeast two-hybrid assay was performed using the ProQuest Two-hybrid System (Invitrogen) according to the manufacturer's instructions. Size exclusion chromatography of SPD-1 full-length proteins was performed on an Äkta system (GE Healthcare) using a Superdex 200 HR 10/30 column (GE Healthcare) in SD150 buffer (150 mM NaCl, 10 mM HEPES (pH 7.7), 1 mM EGTA, 1 mM MgCl_2_, 0.2 mM dithiothreitol).

For microtubule binding, 20 μl of the recombinant full-length SPD-1 purified by Superdex chromatography (0.5 mg ml^−1^ in SD150 buffer) was incubated with 5 μl of taxol-stabilized microtubules (5 mg ml^−1^) and 25 μl of 2 × BRB80 (160 mM PIPES (pH 6.8), 4 mM MgCl_2_, 2 mM EGTA, 2 mM GTP, 2 mM dithiothreitol and 40 μM taxol) supplemented with 40% (v/v) glycerol. Microtubules and bound proteins were collected by ultracentrifugation at 131,000*g* for 20 min using a TLA-100 rotor (Beckman) through a cushion of 1 × BRB80 supplemented with 30% (v/v) glycerol at 25 °C and analysed by SDS–polyacrylamide gel electrophoresis.

Microtubule bundling was assessed by incubating 8 μl of the Superdex-purified SPD-1 (62.5 μg ml^−1^ in SD150 buffer) with 2 μl of taxol-stabilized microtubules (2.5 mg ml^−1^) and 10 μl of 2 × BRB80 supplemented with 40% (v/v) glycerol for 10 min at 25 °C. After fixation with 20 μl of 2% (w/v) glutaraldehyde diluted in 1 × BRB80 supplemented with 20% (v/v) glycerol, the microtubules were sedimented onto a coverglass by ultracentrifugation through layers of 5 ml BRB80 and 2 ml of BRB80 supplemented with 10% glycerol using a SW40 rotor (Beckman) at 217,000*g* for 60 min at 25 °C. The coverglass with sedimented microtubules was fixed with pre-chilled 100% methanol for 10 min at –20 °C and stained for microtubules with DM1a anti-tubulin antibody (Sigma T6199, 1:500 dilution) and Alexa Fluor 488-conjugated anti-mouse secondary antibody (Invitrogen, A11029, 1:2,000 dilution), and observed by an Olympus BX51 microscope equipped with a PlanApo N 60 × /1.42 objective and a CoolSNAP HQ2 CCD camera (Photometrics)^38^.

## Additional information

**How to cite this article:** Lee, K-Y. *et al.* Direct interaction between centralspindlin and PRC1 reinforces mechanical resilience of the central spindle. *Nat. Commun.* 6:7290 doi: 10.1038/ncomms8290 (2015).

## Supplementary Material

Supplementary Information Supplementary Figures 1-7 and Supplementary Table 1.
