# Supplementary material for: Direct interaction between centralspindlin and PRC1 reinforces mechanical resilience of the central spindle
Source: Nat Commun. 2015 Jun 19;6:7290. doi: 10.1038/ncomms8290 (PMC4557309; doi:10.1038/ncomms8290)
Supplement: Supplementary Information — Supplementary Figures 1-7 and Supplementary Table 1. [file ncomms8290-s1.pdf]

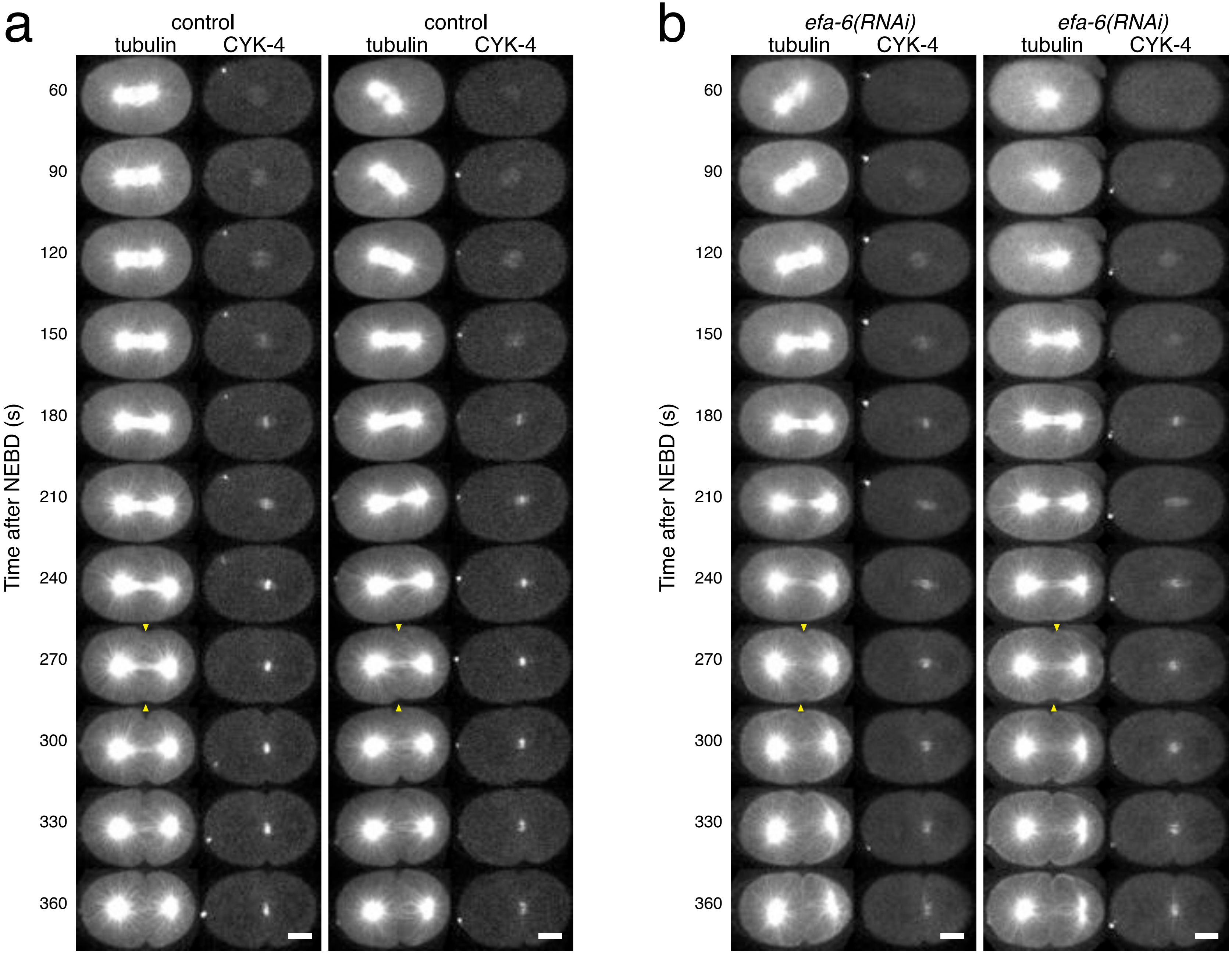

Supplementary Figure 1 Formation and maintenance of the central spindle during the first embryonic division of *C. elegans*.
Embryos expressing mCherry::tubulin and CYK-4::GFP and treated for control RNAi (a) or depleted of EFA-6 (b), BMK-1 (c) or SPD-1 (d) by feeding RNAi were observed by live spinning disk confocal microscopy. Yellow arrowheads indicate the first sign of cleavage furrow ingression. Bar, 10 μm.

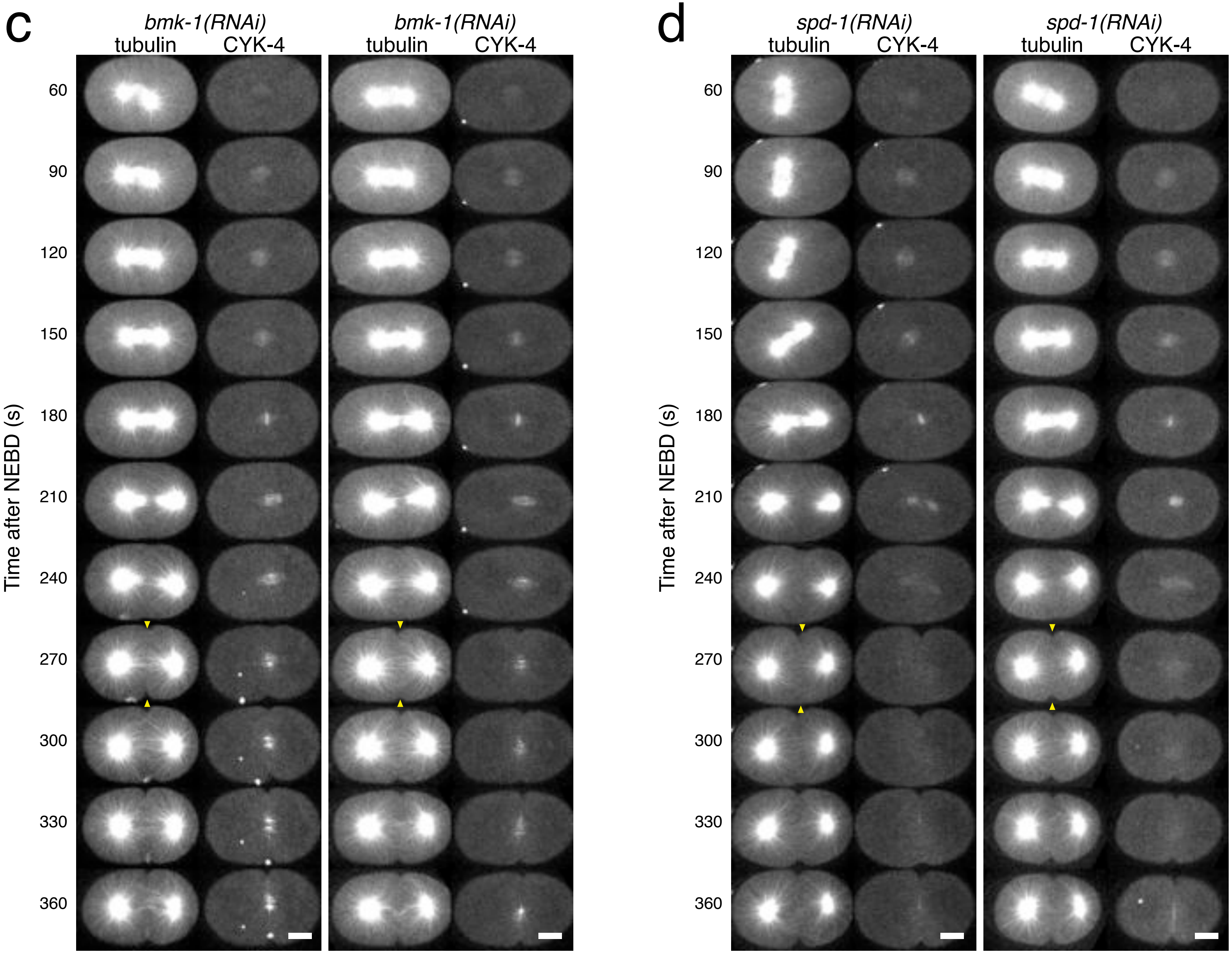

Supplementary Figure 1 Formation and maintenance of the central spindle during the first embryonic division of *C. elegans*. (cont.)

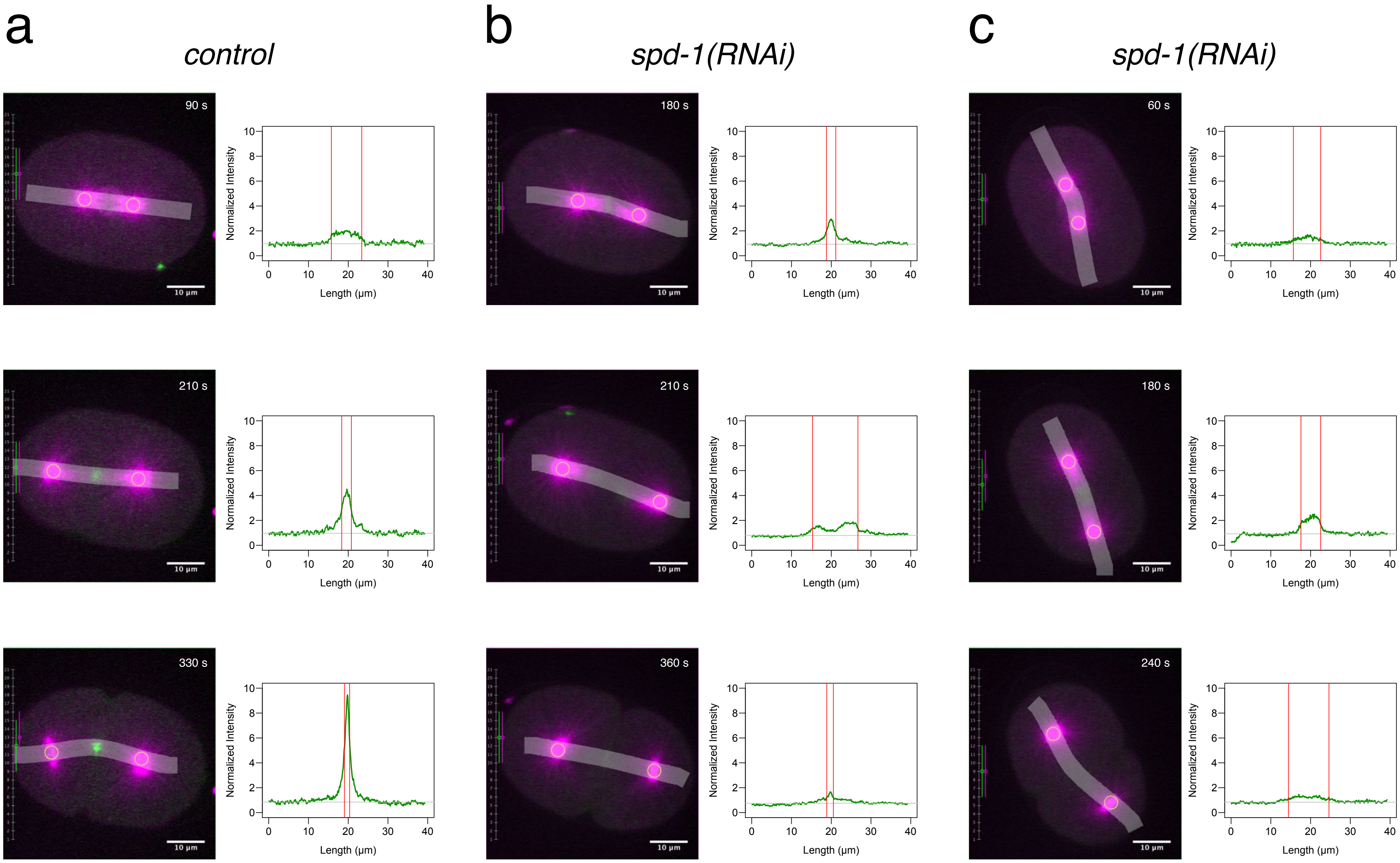

Supplementary Figure 2. Examples of line-profiling of CYK-4::GFP

Average z-projection of the seven best focused slices was made for each channel (green: CYK-4, magenta: tubulin) and time points. Intensity of CYK-4::GFP was measured along a smooth curve (that connects two spindle poles and the peak of CYK-4 and normalized by the mean CYK-4 intensity within the cell above the background level. The graphs show the line profile (green) and basal cellular level (gray) of CYK-4. The positions of 50% of the peak height are indicated in red.

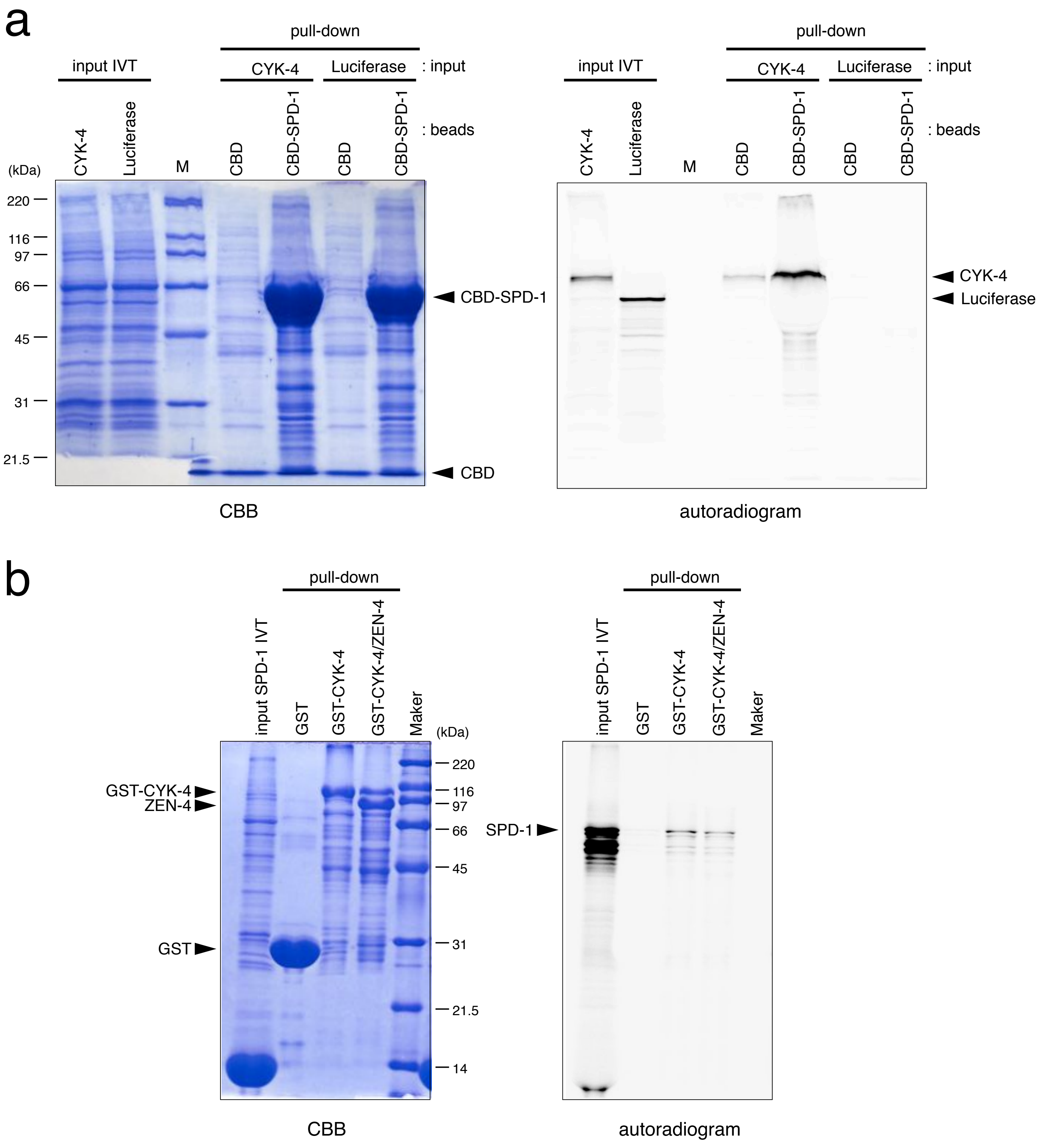

Supplementary Figure 3. Binding between CYK-4 and SPD-1 (Fig. 2a, b full versions)

(a) In vitro translated full-length CYK-4 was pulled down by full-length SPD-1 immobilised on chitin beads via chitin-binding-domain (CBD) tag. (b) In vitro translated full-length SPD-1 was pulled down by full-length CYK-4 or centralspindlin holocomplex (CYK-4/ZEN-4) immobilised on glutathione-sepharose beads via glutathione-S-transferase (GST) tag. The input in vitro translate (IVT) and the proteins bound to the beads were analysed by SDS-PAGE, followed by Coomassie Brilliant Blue staining (CBB) and autoradiography.

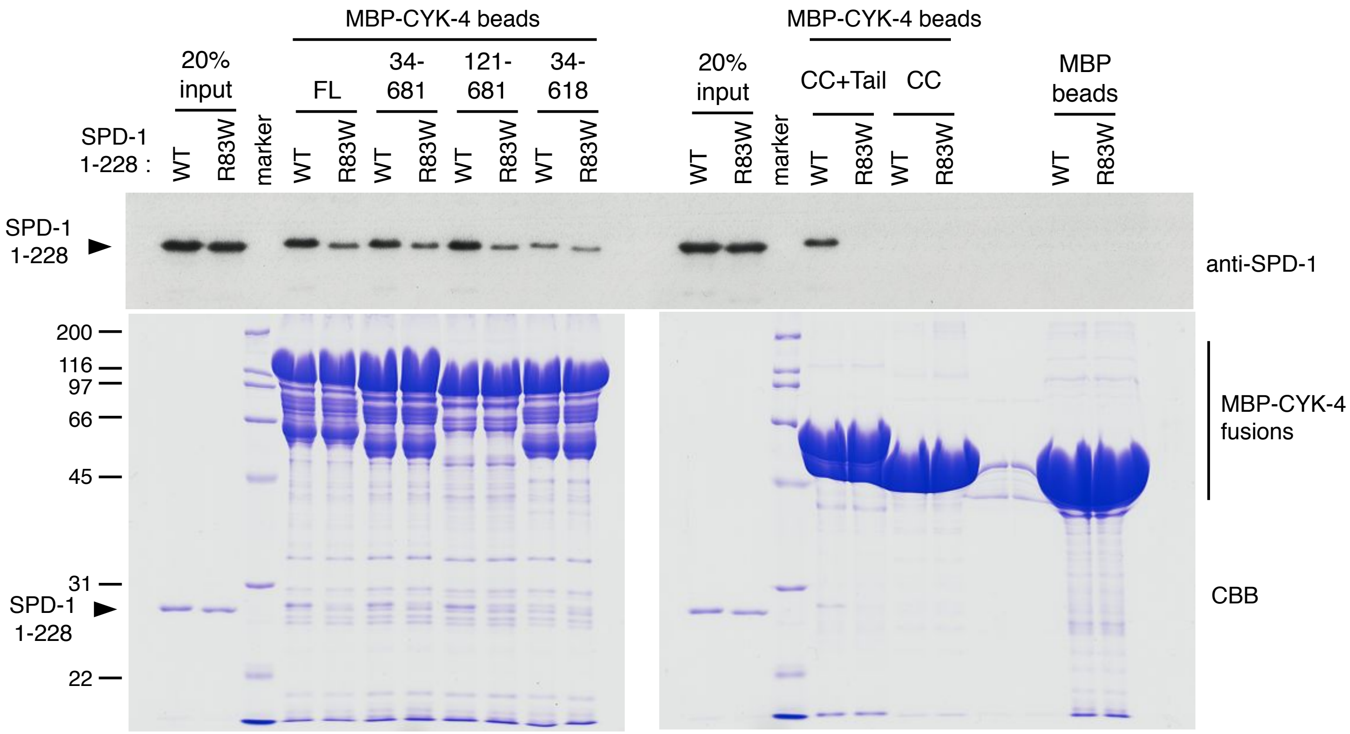

Supplementary Figure 4. Effect of SPD-1 R83W mutation on the SPD-1-CYK-4 interaction (Fig. 2e full version)

SPD-1 1-228 fragment with or without the R83W mutation was pulled down by CYK-4 constructs expressed as fusion proteins with maltose-binding protein (MBP) and detected by western blotting with an anti-SPD-1 antibody. Quality of the beads coated with the CYK-4 constructs was examined by staining with coomassie brilliant blue (CBB).

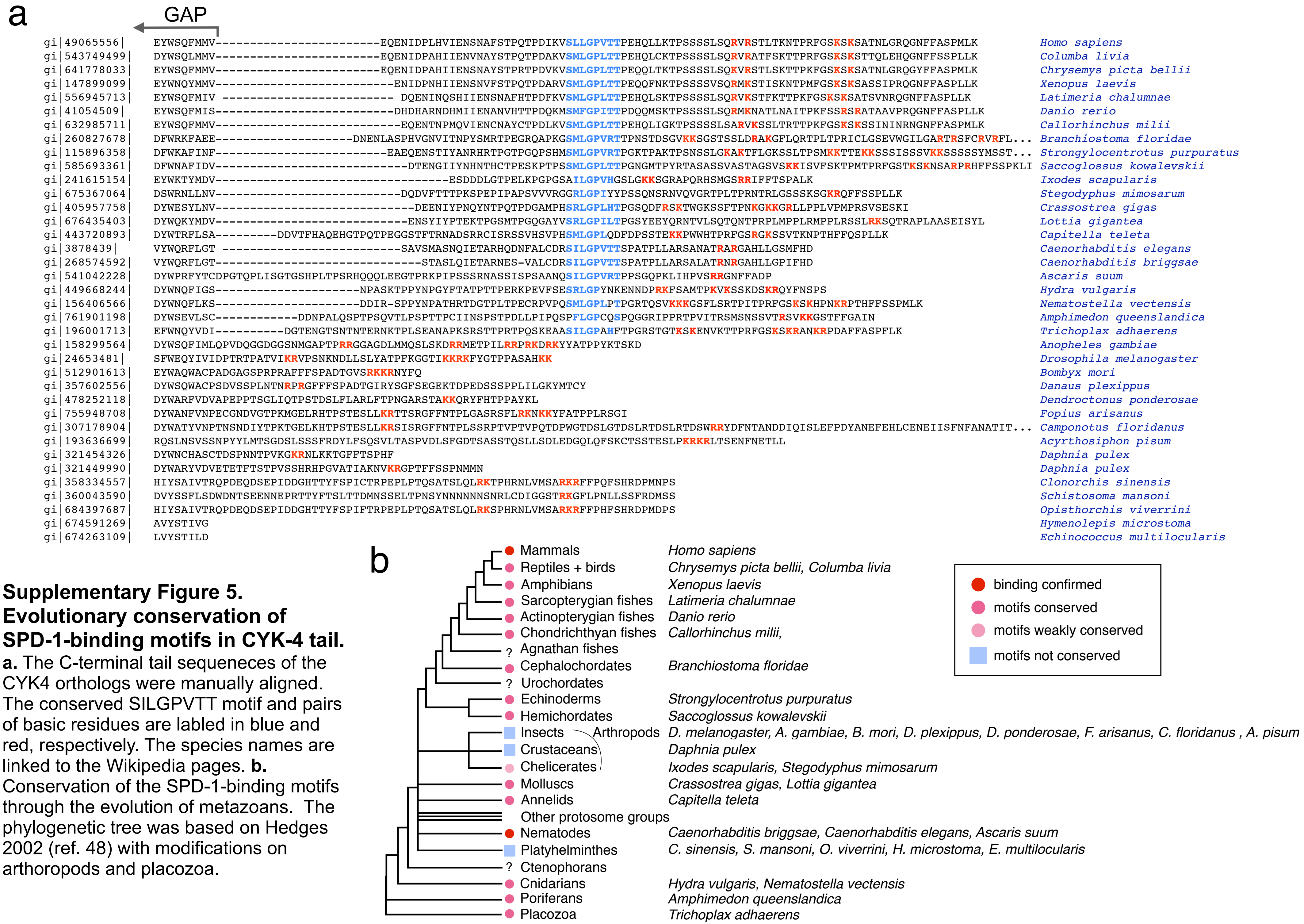

Supplementary Figure 5. Evolutionary conservation of SPD-1-binding motifs in CYK-4 tail.
a. The C-terminal tail sequeneces of the CYK4 orthologs were manually aligned. The conserved SILGPVTT motif and pairs of basic residues are labled in blue and red, respectively. The species names are linked to the Wikipedia pages. **b.** Conservation of the SPD-1-binding motifs through the evolution of metazoans. The phylogenetic tree was based on Hedges 2002 (ref. 48) with modifications on arthoropods and placozoa.

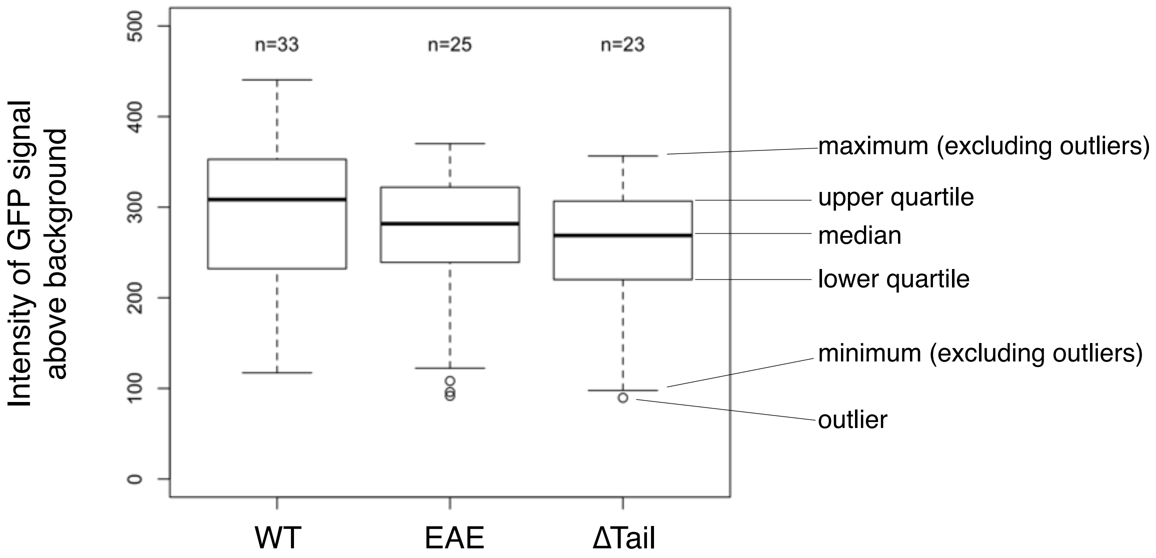

Supplementary Figure 6. Expression levels of *cyk-4::gfp* transgenes

Expression levels of CYK-4::GFP were examined by the fluorescence intensity of GFP. Mean intensity of the uniform CYK-4::GFP signal in the central 6 μm volume (7 slices) of each embryo was measured during the pre-mitotic stage and subtracted by the background signal (outside of the cell). No statistically significant difference between the expression levels was detected. *p*-values of the analysis of variance for all the three combinations of comparison were bigger than 0.3 (after correction for multiple comparison by Turkey).

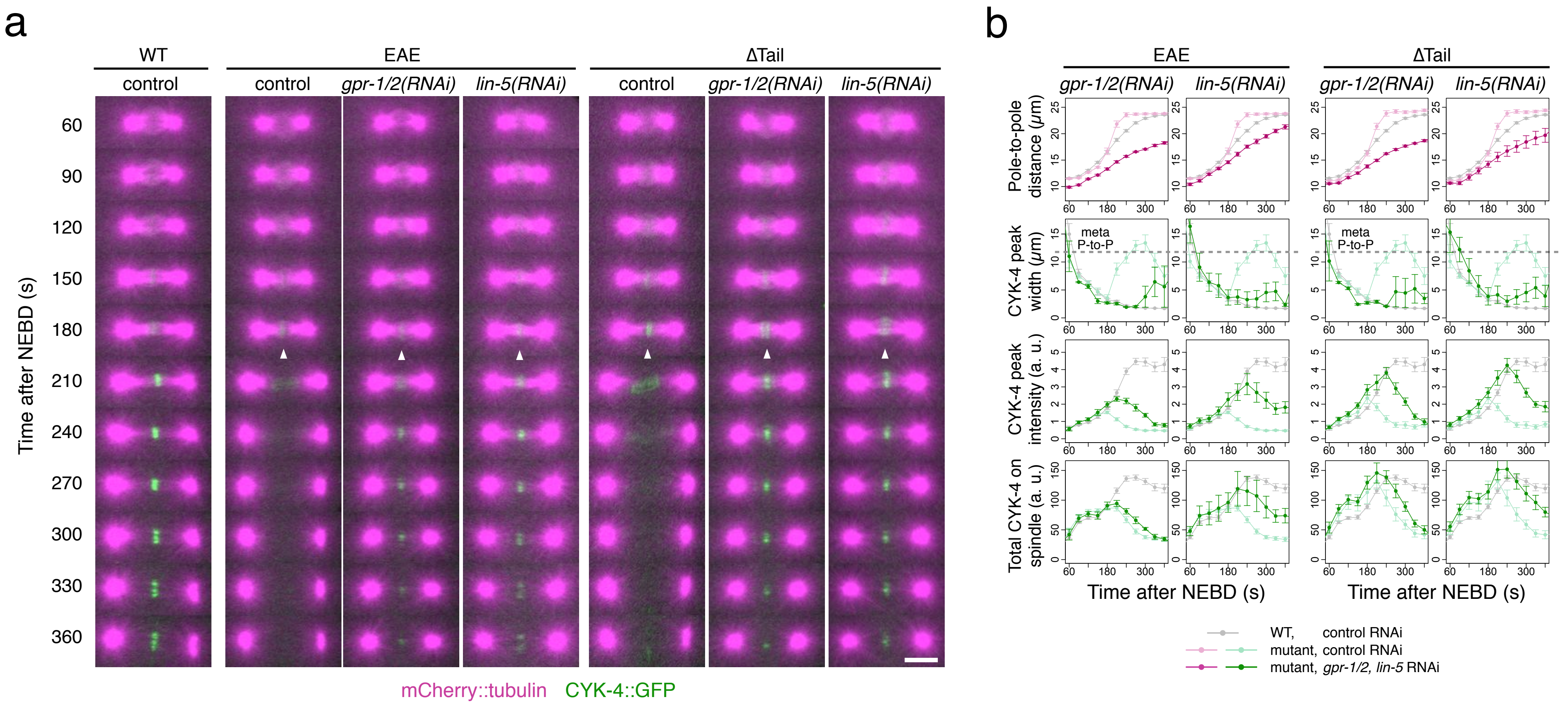

Supplementary Figure 7. Reduction of the cortical pulling forces suppresses the spindle rupture in the EAE mutant embryos

Time lapse imaging of the *cyk-4* EAE and ΔTail mutant embryos depleted of the activators of the cortical pulling force, GPR-1/2 or LIN-5 (n=7 and 12, respectively, for EAE; n=6 and 11, respectively, for ΔTail). Bar, 10 μm.

Supplementary Table 1. List of *C. elegans* strains used in this study

Strain	Genotype
N2	wild type
EG5003	*unc-119(ed3) III; cxTi10882 IV*
JA1559	*weIs21 [pJA138 (pie-1::mCherry::tub::pie-1)]; unc-119(ed3) III*
VC859	*+/mT1 II; cyk-4(ok1034)/mT1[dpy-10(e128)] III*
QM50	*unc-119(ed3) III; xaSi2[cyk-4::gfp cb-unc-119(+)] IV*
QM53	*unc-119(ed3) III; xaSi5[cyk-4CΔ::gfp cb-unc-119(+)] IV*
QM55	*unc-119(ed3) III; xaSi7[cyk-4AA::gfp cb-unc-119(+)] IV*
QM56	*unc-119(ed3) III; xaSi8[cyk-4AA::gfp cb-unc-119(+)] IV*
QM57	*unc-119(ed3) III; xaSi9[cyk-4::gfp cb-unc-119(+)] IV*
QM58	*unc-119(ed3) III; xaSi10[cyk-4EAE::gfp cb-unc-119(+)] IV*
QM59	*unc-119(ed3) III; xaSi11[cyk-4CΔ::gfp cb-unc-119(+)] IV*
QM60	*unc-119(ed3) III; xaSi12[cyk-4EAE::gfp cb-unc-119(+)] IV*
QM71	*+/mT1 II; unc-119(ed3)/mT1[dpy-10(e128)] III; xaSi2[cyk-4::gfp cb-unc-119(+)] IV*
QM72	*+/mT1 II; unc-119(ed3)/mT1[dpy-10(e128)] III; xaSi9[cyk-4::gfp cb-unc-119(+)] IV*
QM73	*+/mT1 II; unc-119(ed3)/mT1[dpy-10(e128)] III; xaSi5[cyk-4CΔ::gfp cb-unc-119(+)] IV*
QM74	*+/mT1 II; unc-119(ed3)/mT1[dpy-10(e128)] III; xaSi11[cyk-4CΔ::gfp cb-unc-119(+)] IV*
QM75	*+/mT1 II; unc-119(ed3)/mT1[dpy-10(e128)] III; xaSi7[cyk-4AA::gfp cb-unc-119(+)] IV*
QM76	*+/mT1 II; unc-119(ed3)/mT1[dpy-10(e128)] III; xaSi8[cyk-4AA::gfp cb-unc-119(+)] IV*
QM77	*+/mT1 II; unc-119(ed3)/mT1[dpy-10(e128)] III; xaSi10[cyk-4EAE::gfp cb-unc-119(+)] IV*
QM78	*+/mT1 II; unc-119(ed3)/mT1[dpy-10(e128)] III; xaSi12[cyk-4EAE::gfp cb-unc-119(+)] IV*
QM79	*+/mT1 II; cyk-4(ok1034)/mT1[dpy-10(e128)] III; xaSi2[cyk-4::gfp cb-unc-119(+)] IV*
QM80	*+/mT1 II; cyk-4(ok1034)/mT1[dpy-10(e128)] III; xaSi9[cyk-4::gfp cb-unc-119(+)] IV*
QM81	*+/mT1 II; cyk-4(ok1034)/mT1[dpy-10(e128)] III; xaSi5[cyk-4CΔ::gfp cb-unc-119(+)] IV*
QM82	*+/mT1 II; cyk-4(ok1034)/mT1[dpy-10(e128)] III; xaSi11[cyk-4CΔ::gfp cb-unc-119(+)] IV*
QM83	*+/mT1 II; cyk-4(ok1034)/mT1[dpy-10(e128)] III; xaSi7[cyk-4AA::gfp cb-unc-119(+)] IV*
QM84	*+/mT1 II; cyk-4(ok1034)/mT1[dpy-10(e128)] III; xaSi8[cyk-4AA:gfp cb-unc-119(+)] IV*
QM85	*+/mT1 II; cyk-4(ok1034)/mT1[dpy-10(e128)] III; xaSi10[cyk-4EAE::gfp cb-unc-119(+)] IV*
QM86	*+/mT1 II; cyk-4(ok1034)/mT1[dpy-10(e128)] III; xaSi12[cyk-4EAE::gfp cb-unc-119(+)] IV*
QM88	*pie-1::mCherry::tub::pie-1; cyk-4(ok1034) III; xaSi2[cyk-4::gfp cb-unc-119(+)] IV*
QM89	*pie-1::mCherry::tub::pie-1; cyk-4(ok1034) III; xaSi11[cyk-4CΔ::gfp cb-unc-119(+)] IV*
QM90	*pie-1::mCherry::tub::pie-1; cyk-4(ok1034) III; xaSi10[cyk-4EAE::gfp cb-unc-119(+)] IV*
QM91	*pie-1::mCherry::tub::pie-1; spd-1(oj5) I; cyk-4(ok1034) III; xaSi2[cyk-4::gfp cb-unc-119(+)] IV*
